# Gut microbiota-derived butyrate and the SIRT1/FoxO1 axis: epigenetic-metabolic regulation of ovarian function in premature ovarian insufficiency—a comprehensive review

**DOI:** 10.3389/fmicb.2026.1856217

**Published:** 2026-07-27

**Authors:** Shengnan Tian, Yang Song, Lixue Liu, Shanshan Chen, Shuxia Wu, Ya Tuo

**Affiliations:** 1 Department of Reproductive Medicine, Affiliated Hospital of Inner Mongolia Medical University, Hohhot, Inner Mongolia, China; 2 Department of Gynecology, The Fifth People's Hospital of Jinan, Jinan, Shandong, China; 3 Key Laboratory of Reproductive Health Diseases Research and Translation of Ministry of Education & Key Laboratory of Human Reproductive Medicine and Genetic Research of Hainan Province & Hainan Provincial Clinical Research Center for Thalassemia, The First Affiliated Hospital of Hainan Medical University, Hainan Medical University, Haikou, Hainan, China

**Keywords:** butyrate, epigenetic reprogramming, gut microbiota, gut-ovary axis, SIRT1/FOXO1

## Abstract

Premature ovarian insufficiency (POI) is a chronic condition affecting approximately 1.1–3.7% of women worldwide. Beyond its primary impact on fertility, POI poses significant long-term health risks, including cardiovascular disease, osteoporosis, and neurocognitive decline. Current clinical interventions, largely limited to hormone replacement therapy, are primarily palliative and do not address the underlying depletion of ovarian reserve. Recent research has identified a potential link between gut microbiota and POI pathogenesis, suggesting that gut–ovary axis dysbiosis may play a pivotal role. Systemic depletion of butyrate-producing microbiota has been shown to induce oxidative stress and granulosa cell apoptosis. In this review, we propose the gut–butyrate–SIRT1–FoxO1 axis as a central theoretical framework. This model advances beyond the conventional “leaky gut–LPS inflammation” model to demonstrate that gut-derived butyrate functions as a trans-organ “metabolic messenger.” Through epigenetic–metabolic coupling, butyrate orchestrates SIRT1–FoxO1 activation. This pathway may mitigate reactive oxygen species (ROS)-induced calcium overload, restore mitochondrial quality control, and preserve granulosa cell homeostasis. Recent preclinical studies have demonstrated that butyrate supplementation can rescue ovarian function in POI models by enhancing SIRT1-mediated FoxO1 deacetylation, This mechanism may suppress pro-apoptotic signaling and promote follicular survival. Furthermore, fecal microbiota transplantation (FMT) from healthy donors has been shown to mitigate ovarian senescence in mice with dysbiotic intestinal microbiota, further substantiating the therapeutic potential of this axis. This review delineates the pleiotropic effects of butyrate across multiple organ systems and provides a robust biological foundation for future microbiota-based interventions. Although substantial experimental validation remains necessary, a deeper understanding of this signaling axis has the potential to transform POI clinical management. Future approaches may shift from palliative, symptomatic treatment to precise disease-modifying therapy.

## 1 Introduction

Premature ovarian insufficiency (POI) is characterized by the loss of ovarian function before the age of 40 years and is diagnosed by persistent amenorrhea (≥4 months), elevated follicle-stimulating hormone (FSH > 25 IU/L), and reduced anti-Müllerian hormone (AMH) levels. The global prevalence of POI is estimated to range from 1.1 to 3.7% (European Society for Human et al., 2016; Golezar et al., 2019). Beyond infertility, POI is associated with increased long-term health risks, including cardiovascular disease, osteoporosis, metabolic disorders, and cognitive decline (Billington et al., 2023; Lumsden et al., 2025). The prevailing clinical management strategy relies on hormone replacement therapy (HRT), which alleviates symptoms but does not reverse the underlying depletion of the ovarian reserve (Erel et al., 2025).

Conventionally, the etiology of POI has been attributed to ovarian pathologies, genetic abnormalities, and endocrine disorders. However, emerging evidence suggests that gut microbiota dysbiosis may act as an upstream driver of ovarian oxidative stress through the gut–ovary axis (Huang et al., 2024). Early mechanistic studies focused predominantly on the “leaky gut” hypothesis. This model proposes that gut microbiota disruption compromises intestinal mucosal barrier integrity, thereby facilitating paracellular translocation of lipopolysaccharide (LPS) from Gram-negative bacteria into the systemic circulation. The resulting endotoxemia has been shown to activate TLR4/NF-κB-signaling, promoting systemic inflammation and oxidative stress that may contribute to premature ovarian reserve exhaustion (He et al., 2022; Liu et al., 2025b; Turner, 2009). Although the LPS-driven inflammatory model provides a plausible explanation for the initiation of ovarian damage, the downstream molecular mechanisms linking gut dysbiosis to ovarian dysfunction remain poorly understood.

**Figure 1:**
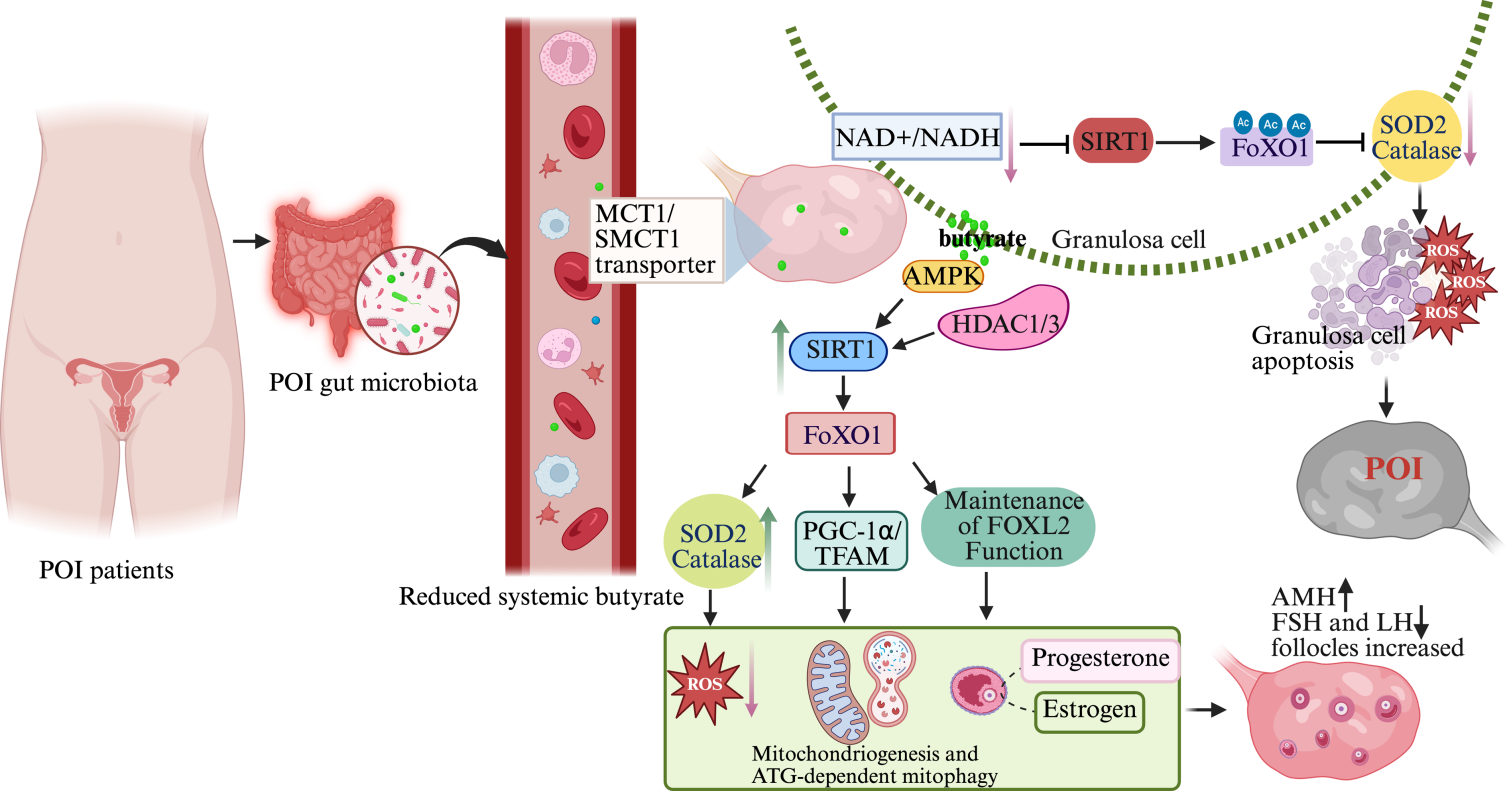
Gut microbiota-derived butyrate protects against premature ovarian insufficiency (POI) via SIRT1/FoxO1-mediated cellular homeostasis. *Pathological axis (upper)*: Gut microbiota dysbiosis in POI patients reduces systemic butyrate, impairing its uptake via MCT1/SMCT1 in granulosa cells. This disrupts SIRT1 signaling and NAD+/NADH balance, causing FoxO1 hyperacetylation. Impaired FoxO1 fails to activate antioxidant genes (SOD2, CAT), leading to ROS accumulation and granulosa cell apoptosis. *Protective axis (lower)*: Adequate butyrate activates AMPK, inhibits HDAC1/3, and upregulates SIRT1. SIRT1-mediated FoxO1 deacetylation promotes nuclear translocation and transcriptional activity, triggering three protective pathways: (1) antioxidant defense (SOD2, CAT upregulation); (2) mitochondrial quality control (PGC-1*α*/TFAM-mediated biogenesis and mitophagy); and (3) granulosa cell identity maintenance (FOXL2 deacetylation). These effects preserve ovarian function, evidenced by elevated AMH, reduced FSH/LH, and increased follicle count. POI, Premature ovarian insufficiency; MCT1/SMCT1, monocarboxylate transporters; SIRT1, Sirtuin 1; FoxO1, Forkhead box O1; AMPK, AMP-activated protein kinase; HDAC, histone deacetylase; SOD2, superoxide dismutase 2; CAT, Catalase; ROS, reactive oxygen species; PGC-1α, peroxisome proliferator-activated receptor gamma coactivator 1-alpha; TFAM, mitochondrial transcription factor A; FOXL2, Forkhead box L2; AMH, anti-Müllerian hormone; FSH, follicle-stimulating hormone; LH, luteinizing hormone. Created with BioRender.com.

High-resolution microbiome profiling has revealed substantial compositional and functional alterations in the gut microbiota of patients with POI, providing population-level evidence for gut–ovary crosstalk (Figure 1). However, the strength and consistency of these associations vary considerably across studies. To enhance interpretive clarity, this review adopts a hierarchical evidence framework. Level 1 includes systematic reviews and meta-analyses of clinical studies, whereas Level 2 discusses clinical cohort studies that involved direct measurement of relevant biomarkers. Level 3 comprises *in vivo* animal models and *in vitro* studies involving cellular models of ovarian or granulosa cell function, while Level 4 includes mechanistic extrapolations from related biological systems. This framework enables a clear distinction between well-established clinical associations and speculative mechanistic models.

Building upon the established “leaky gut–LPS inflammation” paradigm, this review proposes the gut–butyrate–SIRT1–FoxO1 axis as the core mechanistic link between gut microbial ecology and ovarian pathophysiology in POI. Within this framework, gut-derived butyrate is viewed not merely as a local microbial metabolite but also as a trans-organ messenger capable of epigenetically regulating ovarian granulosa cell function. Through activation of SIRT1–FoxO1 signaling, butyrate may regulate antioxidant defense, mitochondrial quality control, and immune remodeling. These effects may collectively influence cell survival. Building on this framework, we discuss an intervention strategy for POI that integrates microbiome restoration, metabolic–epigenetic tuning, and advanced drug delivery approaches. By bridging gut microbial metabolism and ovarian function, this review provides a conceptual foundation for translating emerging mechanistic insights into precision therapeutics for POI.

## 2 From metabolic deficiency to epigenetic silencing: the core pathological cascade in POI

### 2.1 Systemic depletion of butyrate-producing bacteria: a core ecological feature

High-throughput sequencing studies in recent clinical cohorts have demonstrated profound gut microbiota restructuring in patients with POI, characterized by a systemic depletion of butyrate-producing bacteria (Wang et al., 2023; Wu et al., 2021). Specifically, the relative abundances of key butyrate producers within the Firmicutes phylum, including *Faecalibacterium prausnitzii*, *Roseburia* spp., and *Eubacterium hallii*, were significantly reduced in patients with POI compared with healthy controls.

This ecological shift was associated with decreased circulating butyrate levels. The functional consequences of this butyrate depletion extend beyond local intestinal effects. Butyrate deficiency has been reported to compromise intestinal barrier integrity by destabilizing HIF-1α and downregulating tight junction proteins, including ZO-1 and occludin. This change may create a permissive environment for LPS translocation and systemic inflammation (Kelly et al., 2015; Li et al., 2024). However, emerging evidence suggests that the role of butyrate extends beyond intestinal barrier maintenance, with butyrate functioning as a critical metabolic and epigenetic regulator in distal organs, particularly the ovaries.

### 2.2 Butyrate as a trans-organ metabolic messenger

Under physiological conditions, approximately 5–10% of colonic butyrate escapes first-pass hepatic extraction and enters the systemic circulation (Blaak et al., 2020). Its transport across biological barriers is facilitated by monocarboxylate transporter 1 (MCT1; *SLC16A1*) and sodium-coupled monocarboxylate transporter 1 (SMCT1; *SLC5A8*). Multi-omics analyses in mammalian models have detected butyrate in follicular fluid at concentrations reaching 12.56 μmol/L, with levels positively correlated with follicular developmental competence (Xu et al., 2023).

This pharmacokinetic profile supports the concept of butyrate as a bona fide endocrine messenger capable of exerting direct ovarian effects. The concentration gradients from the gut lumen (approximately 10–100 mM) and portal circulation (approximately 100–300 μM) to peripheral tissues (approximately 1–20 μM) provide a physiological basis for the systemic distribution of butyrate.

### 2.3 The SIRT1–FoxO1 axis: a candidate mechanistic node

During butyrate-mediated regulation of granulosa cell fate, the SIRT1–FoxO1 axis has been proposed as a central mechanistic node linking upstream butyrate signaling to downstream cytoprotective effects. This model is supported primarily by Level 3–4 evidence extrapolated from related systems. Within this framework, butyrate-induced activation of SIRT1 promotes the deacetylation of FoxO1 at key lysine residues (Lys242, Lys245, and Lys262), thereby facilitating its nuclear translocation and transcriptional activity (Level 3 evidence) (Brunet et al., 2004; Matsuzaki et al., 2005; Xu et al., 2023).

In the nucleus, deacetylated FoxO1 preferentially binds to promoter regions of antioxidant enzyme genes, including *SOD2*, Catalase, and *GPX1*. In an *in vitro* human ovarian granulosa cell culture model, SIRT1-activated FoxO1 exhibited a 3.2-fold enrichment at the *SOD2* promoter region, resulting in a 2.8-fold increase in transcriptional activity (Ren et al., 2025; Zhou et al., 2025). In animal models of POI, butyrate intervention has been associated with a 42% reduction in the follicular atresia rate and a 35% increase in ovulation number. Notably, while direct evidence from patients with POI remains limited. However, findings from human granulosa cells and animal models collectively support a role for SIRT1/FoxO1 signaling in ovarian antioxidant defense (Level 3–4 evidence) (Feng et al., 2025b; Ren et al., 2025; Wang et al., 2025).

The protective effects of the SIRT1–FoxO1 axis may involve multiple downstream pathways, including antioxidant defense, mitochondrial quality control, immunomodulation, and maintenance of cellular identity. These mechanisms are discussed in greater detail in subsequent sections, where molecular details and supporting experimental evidence are systematically reviewed.

## 3 Remodeling of the ovarian microenvironment by the SIRT1–FoxO1 axis

The protective effects of the SIRT1–FoxO1 axis may extend beyond granulosa cells. These effects may involve the broader ovarian microenvironment through complex intercellular signaling networks. In this context, gut microbial metabolites, particularly butyrate, function as key upstream modulators. They link gut homeostasis to local ovarian immunity. The SIRT1–FoxO1 axis is a crucial target; however, it is important to recognize that butyrate exerts pleiotropic ovarian protective effects through multiple parallel pathways and that the relative contributions of each pathway in the context of POI remain to be fully elucidated.

### 3.1 Multiple parallel pathways of butyrate action

#### 3.1.1 HDAC inhibition and epigenetic remodeling

Butyrate acts as a potent endogenous histone deacetylase (HDAC) inhibitor and induces global epigenetic remodeling independent of SIRT1. This process may promote transcription of genes involved in cell survival and stress responses (Level 3–4 evidence) (Steliou et al., 2012; Waldecker et al., 2008). However, HDAC inhibition exerts broad and relatively nonspecific effects, and the relative contributions of SIRT1-dependent and SIRT1-independent mechanisms to butyrate-mediated ovarian protection remain unclear.

#### 3.1.2 GPCR activation

Butyrate has also been shown to function as a direct ligand for G protein-coupled receptors (GPCRs), particularly FFAR2, FFAR3, and GPR109A. Activation of these receptors may mitigate systemic inflammation and reinforce gut barrier integrity, which may indirectly protect ovarian function (Level 3 evidence) (Brown et al., 2003; Singh et al., 2014; Thangaraju et al., 2009).

#### 3.1.3 Bile acid metabolism

Butyrate-induced alterations in gut microbiota composition may affect secondary bile acid metabolism, potentially suppressing NLRP3 inflammasome activation via receptors such as TGR5 (Level 3–4 evidence) (Guo et al., 2016; Shi et al., 2020). However, direct evidence linking bile acid signaling to ovarian function in POI remains limited.

Collectively, these mechanisms may operate synergistically. Despite this complex network, the SIRT1–FoxO1 axis represents the most extensively characterized mechanistic pathway currently described in POI models and will therefore be the primary focus of subsequent sections.

### 3.2 Immunoregulatory modulation

At the immunoregulatory level, SIRT1 has been shown to downregulate the transcriptional activity of pro-inflammatory factors such as TNF-α and IL-6 through inhibition of NF-κB (p50/p65) nuclear translocation. This action simultaneously reduces the inflammatory effects of LPS-induced TLR4 signaling in ovarian tissue (Level 2–3 evidence) (He et al., 2022; Yang et al., 2022).

This anti-inflammatory effect may be closely associated with M2 macrophage polarization and maintenance of immune tolerance, potentially reducing persistent inflammatory injury to granulosa cells (Yang et al., 2022). Gut microbiota homeostasis is important for regulating this process. Specific probiotic strains may restore intestinal barrier integrity and reduce paracellular LPS translocation, thereby potentially limiting systemic inflammatory responses. The subsequent effects of microbial metabolites, including butyrate, on local ovarian immune homeostasis may be mediated, at least in part, through SIRT1 signaling pathways (Level 3 evidence) (Zhao et al., 2024). Furthermore, single-cell sequencing data from ovarian tissues of patients with POI revealed a significant positive correlation between SIRT1 activity and the proportions of regulatory T cells (Tregs) (*r* = 0.78, *p* < 0.001). These findings suggest that SIRT1–FoxO1 signaling may contribute not only to local anti-inflammatory effects but also to systemic immune homeostasis (Gomes et al., 2013).

### 3.3 Epigenetic maintenance of cellular identity

At the cellular network level, SIRT1 activation may contribute to the epigenetic maintenance of granulosa cell identity. SIRT1-mediated FoxO1 deacetylation exerts a dual function by blocking pro-apoptotic transcriptional programs and preventing entry into premature inflammatory exhaustion states by limiting p53 signaling (Level 3 evidence) (Benayoun et al., 2009, 2011; Park et al., 2021; Pavlová et al., 2013). Consequently, granulosa cells may recover from oxidative damage-induced functional suppression and reestablish normal physiological phenotypes characterized by steroid hormone synthesis.

This maintenance of cellular identity ensures continued granulosa cell sensitivity to gonadotropin signals, thereby limiting the pathological processes underlying follicular atresia at the transcriptional level. In summary, the SIRT1–FoxO1 axis functions as a molecular nexus that regulates granulosa cell resistance to POI-related pathological processes. This regulation integrates three major pathways: antioxidant defense, mitochondrial quality control, and immune microenvironment remodeling (Guo et al., 2022; Li et al., 2023). The clinical relevance of the SIRT1–FoxO1 axis lies in its potential to provide actionable molecular targets for precision therapeutic strategies targeting gut metabolism–epigenetic networks.

## 4 Mechanisms of butyrate-mediated ovarian protection

### 4.1 Breaking the ROS-calcium overload vicious cycle

In a compromised ovarian microenvironment, exogenous toxins or endogenous metabolites can trigger reactive oxygen species (ROS) surges. These ROS changes promote calcium release from the endoplasmic reticulum and mitochondrial calcium accumulation, resulting in intracellular “calcium overload.” This process directly activates calpain and synergizes with Bax/Bak to increase mitochondrial outer membrane permeability (MOMP). It also stimulates the tricarboxylic acid cycle, which further increases ROS production and reinforces a self-amplifying “ROS-calcium-ROS” vicious cycle (Gielecińska et al., 2024; Ren et al., 2025).

In response to this cascade, the butyrate-driven SIRT1/FoxO1 signaling axis may exert multifaceted protective effects. First, at the transcriptional level, SIRT1-mediated deacetylation remodels FoxO1 conformation. This change promotes FoxO1 recruitment to the promoter regions of antioxidant genes such as *SOD2* and Catalase (Brunet et al., 2004; Ren et al., 2025). FoxO1-mediated transcriptional activation may establish a robust redox barrier within granulosa cells, thereby limiting sustained ROS accumulation.

Restoration of redox homeostasis may be particularly significant because it can protect mitochondrial function. In oxidative stress-induced microenvironmental disorders, the structural integrity of mitochondria-associated endoplasmic reticulum membranes (MAMs) is severely disrupted, contributing to pathological calcium leakage. SIRT1 reactivation may help maintain mitochondrial membrane potential (ΔΨm), which may reduce the risk of mitochondrial permeability transition pores (mPTP) and subsequent caspase-mediated apoptotic cascades (Level 3 evidence) (An et al., 2025; Wang et al., 2025). Additionally, it may support oocyte quality by facilitating gap junction-mediated nicotinamide mononucleotide (NMN) transfer for precise meiotic spindle assembly (Ma et al., 2019). These findings are primarily derived from cellular models of oxidative stress and require further validation in primary granulosa cells and animal models of POI.

### 4.2 Mitochondrial quality control and restoration of bioenergetic resilience

Butyrate-induced SIRT1 reactivation has been shown to counteract metabolic dysfunction associated with NAD + depletion and excessive Poly(ADP-ribose) Polymerase (PARP) activation (An et al., 2024; Bai et al., 2025). This process may potentially reverse SIRT1 enzymatic silencing driven by substrate scarcity. Furthermore, butyrate has been shown to promote an epigenetic shift in granulosa cells from a metabolically compromised state toward a more adaptive phenotype. This homeostatic reconstruction is dependent on the coordinated regulation of mitochondrial biogenesis and autophagy via the SIRT1–FoxO1 axis.

Nuclear-localized SIRT1 directly activates the metabolic master regulator PGC-1α through deacetylation, which subsequently promotes the upregulation of mitochondrial transcription factor A (TFAM). Although the activation of this pathway facilitates mitochondrial biogenesis at the transcriptional level, direct evidence in ovarian granulosa cells remains limited, with current support derived largely from studies in other metabolically active tissues (Feng et al., 2025a; Guo et al., 2022; Li et al., 2023). This process is crucial for meeting the high ATP demand associated with steroid synthesis and oocyte meiosis.

In addition, this signaling axis collaborates with SIRT3 to enhance respiratory chain activity and reduce electron leakage, thereby limiting endogenous ROS production (Chen et al., 2024; Pan et al., 2022; Xu et al., 2023). Furthermore, the SIRT1–FoxO1 axis coordinates with ATG3-mediated mitophagy pathways to facilitate the selective removal of damaged mitochondria. This process may reduce cellular apoptosis and ferroptosis progression (Singh and Ubaid, 2020; Zhao et al., 2020). These processes function synergistically; SIRT1–FoxO1 promotes *de novo* biogenesis of healthy mitochondria while also facilitating ATG-mediated mitophagic clearance of dysfunctional mitochondria. Together, these mechanisms may contribute to the restoration of mitochondrial homeostasis in granulosa cells.

### 4.3 SIRT1-mediated FOXL2 deacetylation maintains granulosa cell identity

Furthermore, SIRT1-mediated deacetylation has been reported to extend to post-translational modifications of the ovary-specific transcription factor FOXL2. Current evidence is primarily based on evidence from cellular models of ovarian granulosa cell function and ovarian cancer-derived cell lines (Benayoun et al., 2011; Zhang et al., 2016). In the context of POI, this modification may prevent premature inflammatory senescence and cell cycle arrest. Consequently, granulosa cells may transition from damage-response states to functional secretory phenotypes dominated by steroid hormone synthesis (Benayoun et al., 2009, 2011).

The maintenance of cellular identity ensures continued granulosa cell sensitivity to gonadotropin signals, which may limit follicular atresia through transcriptional regulation (Li et al., 2020). This butyrate-induced intracellular repair may ultimately result in increased levels of ovarian reserve markers, including AMH and greater follicle abundance (Guo et al., 2022; Li et al., 2023). Simultaneously, it may optimize the oocyte developmental environment through gap junctions, establishing a biological foundation for restoring female reproductive potential (Alam et al., 2021; Han et al., 2017).

### 4.4 Contradictory evidence and context-dependent effects

Although the SIRT1–FoxO1 axis is broadly regarded as pro-survival, the effect of SIRT1 is highly context-dependent. In ovarian cancer, SIRT1 has been shown to play a multifaceted role, acting as a tumor promoter or suppressor depending on the cellular context and tumor microenvironment (Ding et al., 2025). SIRT1-mediated deacetylation also promotes cell survival, proliferation, and metastasis in ovarian cancer, which may contribute to tumor progression. Furthermore, elevated SIRT1 expression correlates with poor prognosis, aggressive tumor behavior, and chemotherapy resistance. High SIRT1 levels are also associated with reduced overall survival and increased tumor grade and stage (Ding et al., 2025). These findings underscore the need to consider SIRT1 modulation within a specific pathological context rather than assuming that it is universally beneficial.

Moreover, the relationship between SIRT1 and its targets, including FoxO1, p53, and PGC-1α, is bidirectional. The AMPK-SIRT1-PGC-1α axis operates through a sophisticated positive feedback loop. AMPK activation elevates intracellular NAD + levels and promotes SIRT1 activity. Activated SIRT1 deacetylates PGC-1α, driving mitochondrial biogenesis and function, which may further reinforce SIRT1 activity (Chen et al., 2025). However, under conditions of severe NAD + depletion, which is common in advanced POI, this feedback may become dysfunctional. Consequently, pharmacological SIRT1 activation alone may be insufficient without concurrent NAD + replenishment.

### 4.5 Dose-dependent effects and safety considerations

Notably, the beneficial effects of butyrate are dose- and context-dependent. Its immunomodulatory mechanism is complex, as its effects on immune function are strongly influenced by concentration. Low concentrations of butyrate (0.2–1 mM) have been shown to inhibit IL-8 release and suppress inflammatory responses, whereas higher concentrations (20–30 mM) increase IL-8 expression and secretion, which may exert pro-inflammatory effects (Liu et al., 2025a). Physiological concentrations (1–5 mM) are associated with pro-survival effects in granulosa cells. In contrast, higher concentrations may induce cell cycle arrest and, in extreme cases, apoptosis via HDAC inhibition (Waldecker et al., 2008).

Furthermore, chronic systemic administration of high-dose butyrate regimens has been associated with gastrointestinal discomfort, altered stool consistency, and potential disruption of gut microbial balance through selective ecological pressure on non-butyrate-producing taxa. High butyrate concentrations may also stimulate epithelial cell apoptosis and disrupt intestinal barrier function (Gerunova et al., 2024). Therefore, these safety considerations should be carefully evaluated in future translational studies.

## 5 Therapeutic strategies targeting the gut–butyrate–SIRT1/FoxO1 axis

The translational potential of the gut–butyrate–SIRT1–FoxO1 axis has prompted the development of multiple intervention strategies that target different nodes of this pathway. These strategies can be stratified into a three-tiered framework based on translational maturity.

### 5.1 Level 3: clinically established strategies include dietary modification and microbial restoration

Dietary intervention currently represents the most accessible and clinically feasible modality for modulating the gut-ovary axis. Clinical studies have shown that dietary approaches selectively enrich core butyrate-producing bacteria, including *Faecalibacterium prausnitzii*, *Roseburia* spp., and *Eubacterium hallii*, by supplying sufficient fermentation substrates. These interventions have also been associated with a reduction in systemic inflammatory markers (Gill et al., 2021; Liu et al., 2025; Wang et al., 2021). Evidence from clinical cohorts with metabolic or reproductive disorders has supported dietary modification as a clinically viable strategy for restoring microbial ecology. However, these benefits are contingent on patients’ baseline gut microbiota composition, and dietary interventions alone may be ineffective in cases of severe dysbiosis.

For patients with refractory or severe microbial dysbiosis, FMT has emerged as a more direct means of rebuilding the gut ecosystem. Previous studies have demonstrated that FMT can restore butyrate-producing bacteria and improve reproductive parameters in animal models of ovarian insufficiency (Davar et al., 2021; Smits et al., 2013; Suez et al., 2018). Its translational potential is further supported by emerging evidence from clinical trials under related conditions. Specific probiotic strains such as *Limosilactobacillus reuteri* and their postbiotic metabolites, including *β*-resorcylic acid, have been shown to function as trans-organ messengers. These signals may modulate oxidative apoptotic cascades in granulosa cells (Feng et al., 2024). These findings suggest that the proposed strategy may serve as an adjunct or alternative treatment for restoring ovarian function.

### 5.2 Level 2: early clinical exploration of SIRT1-targeting pharmacological interventions

SIRT1 represents a key druggable node within the gut–butyrate–SIRT1–FoxO1 axis, making it a promising target for pharmacological interventions in POI. Several metabolic modulators with established safety profiles in other clinical indications have been shown to activate SIRT1 signaling in preclinical models of POI. For example, metformin, a medication commonly prescribed for metabolic conditions, has been reported to reduce cisplatin-induced ovarian insufficiency by activating the SIRT1/Nrf2 pathway (Feng et al., 2025b; Zheng et al., 2025). Similarly, protocatechuic acid has demonstrated protective effects against chemotherapy- or endocrine-disruptor-induced ovarian decline through SIRT1-dependent mechanisms (Feng et al., 2025b; Zheng et al., 2025). These compounds have already received regulatory approval for other uses, which could facilitate their repurposing for future POI clinical trials. The full landscape of these therapeutic strategies and their current evidence levels are summarized in Table 1.

**Table 1:** Therapeutic interventions targeting the SIRT1 axis in ovarian protection.

Category	Intervention	Study model	Molecular mechanisms/key targets	Major findings	Translational stage	References
SIRT1 pathway modulators						
	Protocatechualdehyde	Cyclophosphamide-induced POI in Wistar rats	Activates SIRT1/p53 signaling axis; inhibits excessive granulosa cell apoptosis and senescence	Significantly protects ovary from chemotherapy-induced damage; improves key POI-related clinical and histological indicators	Preclinical	Feng et al. (2025b)
	Metformin	Cisplatin-induced ovarian injury in human granulosa cells & POI animal models	Activates SIRT1/Nrf2 signaling pathway; enhances endogenous antioxidant defense system	Effectively attenuates cisplatin-induced ovarian toxicity; prevents chemotherapy-associated POI progression	Clinical (repurposed)	Zheng et al. (2025)
Microbial metabolites & natural products						
	Oral oyster polypeptides	D-galactose-induced POI in C57BL/6 mice	Exerts natural antioxidant and anti-aging effects via SIRT1 activation	Effectively counteracts D-galactose-induced POI symptoms; preserves overall ovarian function	Preclinical	Li et al. (2020)
	β-Resorcylic acid (Limosilactobacillus reuteri-derived)	Cisplatin-induced ovarian toxicity/infertility mouse models	Bioactive microbial metabolite with direct anti-inflammatory and antioxidant properties	Prevents cisplatin-induced ovarian damage and preserves fertility *in vivo*	Preclinical	Feng et al. (2024)
	Short-chain fatty acids (SCFAs, including butyrate)	Chemotherapy-induced POI mouse models	Multimodal actions: energy substrates, immune modulation, GPCR signaling, epigenetic regulation	Essential for maintaining ovarian homeostasis and protects follicular reserve from injury	Preclinical	Blaak et al. (2020)
Targeted delivery & regenerative therapies						
	Growth hormone (GH) via FSHR-targeted nanocarriers	POI animal models/preclinical studies	Nanocarrier-mediated targeted delivery to dominant follicles	Achieves precise GH enrichment in ovarian tissue; significantly improves local bioavailability	Preclinical	Yang et al. (2025)
	MSCs & MSC-derived extracellular vesicles (EVs) with targeted modification	Damaged ovary models/preclinical studies	Paracrine effects via growth factors, cytokines, and functional miRNAs	Effectively repairs and remodels damaged ovarian microenvironment and function	Clinical trial phase	Song et al. (2024), Yang et al. (2025)
Microbiota modulation strategies						
	Autologous fecal microbiota transplantation (FMT)	Gut mucosal microbiome reconstruction models	Restores beneficial microbial abundance and function; corrects post-antibiotic dysbiosis	Safely and effectively reconstructs intestinal mucosal microbiome to recover butyrate production	Clinical	Suez et al. (2018)
	Allogeneic fecal microbiota transplantation (FMT)	Immunotherapy-resistant cancer patients with gut dysbiosis	Modulates gut microbiota composition; enhances immune cell activation in tumor microenvironment	Significantly improves immunotherapy efficacy via systemic gut-immune-ovary axis crosstalk	Clinical	Davar et al. (2021)
	Autochthonous fecal viral transfer (FVT)	Antibiotic-perturbed murine gut microbiome models	Viruses modulate bacterial abundance, diversity, and ecological function	Promotes efficient recovery of gut microbiota diversity and function post-antibiotic depletion	Preclinical	Draper et al. (2020)
Butyrate & derivatives (repurposed in other metabolic diseases)						
	Sodium butyrate	Diabetic nephropathy rat models	Upregulates AMPK/SIRT1/PGC-1α signaling pathway	Significantly ameliorates renal injury via SIRT1 activation; provides mechanistic reference for ovarian protection	Clinical (repurposed)	Chen et al. (2025)
	Oral tributyrin (butyrate prodrug)	Rat liver inflammation models	Tributyrin slowly releases butyrate in vivo; suppresses NF-κB inflammatory pathway	Effectively elevates circulating butyrate levels; protects tissues from inflammatory injury	Preclinical	Miyoshi et al. (2011)
	Tributyrin + active vitamin D combination	Human colon cancer cell lines	Butyrate inhibits HDAC activity; vitamin D regulates downstream gene expression via VDR	Combination therapy significantly enhances anti-proliferative effects; demonstrates synergistic benefit	Preclinical	Gaschott et al. (2001)

**Figure 2:**
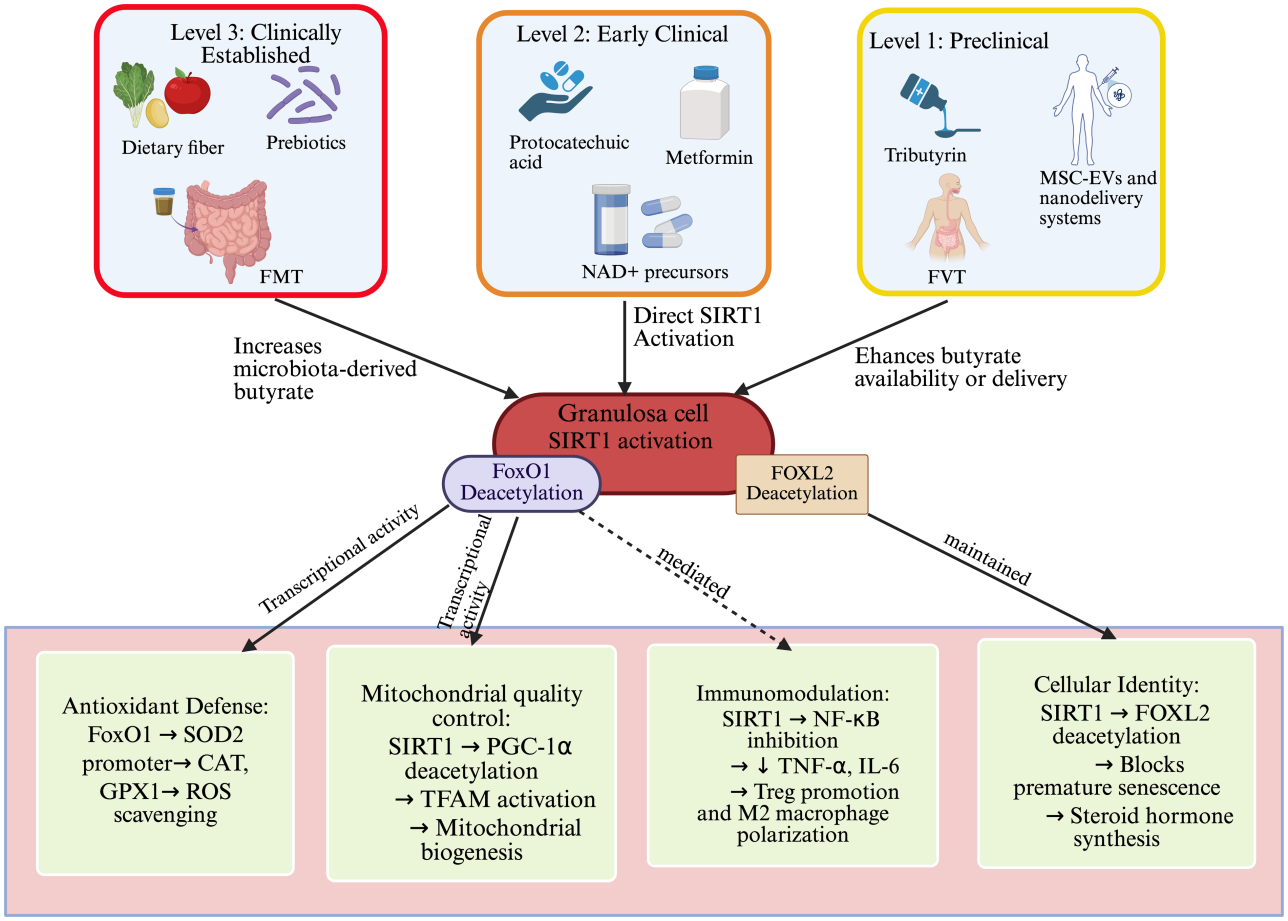
Integrated therapeutic strategies targeting the gut-butyrate-SIRT1/FoxO1 axis in premature ovarian insufficiency (POI). *Level 3* (Clinically Established): High-fiber diet, fecal microbiota transplantation (FMT), and probiotics enrich butyrate-producing gut bacteria (*Faecalibacterium prausnitzii*, *Roseburia* spp.*, Eubacterium hallii*), increasing endogenous butyrate production. *Level 2* (Early Clinical Exploration): SIRT1-targeting agents, including metformin (SIRT1/Nrf2 activation), protocatechuic acid (SIRT1-dependent signaling), and NAD^+^ precursors (nicotinamide mononucleotide [NMN]/nicotinamide riboside [NR]), directly activate the SIRT1/FoxO1 axis. *Level 1* (Preclinical Validation): Advanced delivery platforms, including tributyrin prodrugs (plasma esterase cleavage extends half-life), FSHR-targeted nanocarriers (bypass blood-follicle barrier), mesenchymal stem cell-derived extracellular vesicles (MSC-EVs), and fecal virome transplantation (FVT), overcome pharmacokinetic limitations of free butyrate. *Central mechanism*: Activated SIRT1 deacetylates FoxO1 and FOXL2, promoting downstream protective effects in ovarian granulosa cells: (1) Antioxidant defense via SOD2, CAT, and GPX1 upregulation; (2) Mitochondrial quality control through PGC-1α/TFAM-mediated biogenesis and mitophagy; (3) Immunomodulation via NF-κB inhibition (↓TNF-α, IL-6) and Treg/M2 macrophage promotion; and (4) Maintenance of granulosa cell identity through FOXL2-mediated steroidogenesis. Solid arrows, direct effects; dashed arrows, indirect mediation. FMT, fecal microbiota transplantation; NMN, nicotinamide mononucleotide; NR, nicotinamide riboside; MSC-EVs, mesenchymal stem cell-derived extracellular vesicles; FVT, fecal virome transplantation; SIRT1, sirtuin 1; FoxO1, forkhead box O1; FOXL2, forkhead box L2; SOD2, superoxide dismutase 2; CAT, catalase; GPX1, glutathione peroxidase 1; PGC-1α, peroxisome proliferator-activated receptor gamma coactivator 1-alpha; TFAM, mitochondrial transcription factor A; NF-κB, nuclear factor kappa B; TNF-α, tumor necrosis factor alpha; IL-6, interleukin-6; Treg, regulatory T cell. Created with BioRender.com.

Moreover, the combined administration of butyrate derivatives and NAD + precursors has shown potential synergistic effects in pharmacological studies. This combination may be effective in relieving epigenetic inhibition and simultaneously replenishing the SIRT1 coenzyme substrate. As illustrated in Figure 2, butyrate-mediated SIRT1–FoxO1 activation may enhance the functional capacity of the downstream antioxidant network. The combination of butyrate derivatives with NMN or nicotinamide riboside (NR) warrants further investigation as a dual-track therapeutic approach.

### 5.3 Level 1: preclinical validation involving targeted delivery engineering

A major impediment to butyrate-based therapy advancement is pharmacokinetic limitation. Free butyrate is extensively metabolized in the liver during its initial passage through the gastrointestinal tract, which leads to substantial loss and limited delivery to ovarian tissue (Hamer et al., 2008; Pituch et al., 2013). Although prodrug modification strategies such as tributyrin can increase the *in vivo* half-life via plasma esterase-mediated cleavage (Gaschott et al., 2001; Miyoshi et al., 2011), more transformative solutions are anticipated to emerge from advances in nanodelivery engineering.

Emerging active-targeting platforms, including follicle-stimulating hormone receptor (FSHR)-targeted-peptide-conjugated nanocarriers and mesenchymal stem cell-derived extracellular vesicles, offer promising approaches for ovarian-specific drug delivery (Song et al., 2024; Yang et al., 2025; Zhang et al., 2013). The fundamental design objective of these systems is to traverse the blood-follicle barrier and achieve granulosa cell enrichment, which may minimize the systemic off-target risks associated with high-dose pan-HDAC inhibition. Furthermore, fecal virome transplantation (FVT) represents a next-generation microecological strategy. It targets underlying phage-bacterial interaction networks within the gut and may have potential applications in chemotherapy-induced POI. However, its validity is currently limited to animal models, and significant safety and technical issues remain unresolved (Draper et al., 2020).

### 5.4 Integrated translational outlook

Clinical translation of the gut–butyrate–SIRT1–FoxO1 axis requires a staged, multidimensional approach. Dietary and microbial strategies form the basis of this research (Level 3), whereas pharmacological SIRT1 modulators offer an intermediate translational potential (Level 2). Advanced delivery platforms represent an emerging frontier in precision medicine (Level 1). A closed-loop strategy that integrates microecological restoration, epigenetic-metabolic modulation, and active targeted delivery may transform POI management from palliative hormone replacement to disease-modifying approaches.

However, the inherent limitations of this framework must be acknowledged. Elevated butyrate concentrations have been shown to impede cell proliferation under specific conditions, and SIRT1 function is recognized as context-dependent, varying according to cell type and environment. Consequently, systematic dose–response and cell-specific studies will be essential to establish safety boundaries and optimize the therapeutic window of these interventions prior to clinical translation.

## 6 Discussion

### 6.1 Clinical feasibility and translational barriers

This review proposes the “gut–butyrate–SIRT1–FoxO1 axis” as a conceptual framework that highlights the central role of gut-derived butyrate as a cross-organ metabolic messenger in reversing epigenetic silencing and NAD + metabolic dysfunction in granulosa cells. In addition, it examines its contribution to restoring ovarian antioxidant defenses. Despite the therapeutic rationale supporting this strategy, its clinical translation faces several challenges that warrant rigorous and objective evaluation to avoid overestimating its current applicability.

The translation of gut–ovary axis-targeted interventions is hampered by substantial technical and biological barriers. Direct microbiome remodeling often produces substantial clinical heterogeneity. For example, the efficacy of high-fiber diets and probiotic interventions for increasing butyrate production depends on the patient’s baseline enterotype and systemic metabolic network (Arumugam et al., 2011). Furthermore, temporal discrepancies between microbial remodeling and the potentially narrow therapeutic window in rapidly progressive POI remain a concern. Regarding direct ecological interventions, FMT is associated with risks of incidental infections and bacteremia (Marcella et al., 2021). Inter-donor compositional heterogeneity remains a major obstacle to standardizing FMT protocols (Cammarota et al., 2019). In women of reproductive age, FMT also introduces theoretical risks of pathogen transmission and requires stringent regulatory approval, including investigational new drug (IND) or clinical trial application (CTA) authorization. Evaluation of the actual clinical benefits of these strategies depends on high-quality randomized controlled trials (RCTs) stratified by baseline microbiome profiles.

Regarding pharmacological delivery, butyrate exhibits inherent pharmacokinetic limitations. Free butyrate undergoes rapid absorption in the proximal colon and extensive hepatic first-pass metabolism, which may result in limited ovarian exposure after conventional administration (Hamer et al., 2008; Pituch et al., 2013). Prodrug modifications, such as tributyrin, have been shown to extend the circulating half-life to some extent (Conley et al., 1998; Edelman et al., 2003); however, maintaining therapeutically relevant intraovarian concentrations without triggering systemic off-target HDAC inhibition remains a methodological bottleneck (De Souza et al., 2020). The development of innovative delivery systems, including mesenchymal stem cell-derived extracellular vesicles (MSC-EVs) and targeted nanocarriers, may help overcome these pharmacokinetic barriers. However, clinical translation of advanced therapy medicinal products (ATMPs) requires addressing engineering and toxicological challenges, including scalable Good Manufacturing Practice (GMP)-compliant production, batch-to-batch reproducibility, and immunogenicity associated with repeated allogeneic administration (Herrmann et al., 2021; Song et al., 2024; Yang et al., 2025).

Furthermore, long-term safety evaluation of interventions targeting the SIRT1–FoxO1 axis and their potential systemic endocrine effects is essential. SIRT1 function is highly cell-type specific and microenvironment dependent; therefore, systemic pan-SIRT1 activation may cause off-target effects in non-target tissues (Imai and Guarente, 2014; Liu et al., 2013). Additionally, the pharmacokinetic interactions between SIRT1 modulators and concurrent HRT in patients with POI remain to be defined. From a systems biology perspective, artificial restructuring of the gut microbiome may interfere with endogenous estrogen metabolism (Plottel and Blaser, 2011), which could disrupt the feedback homeostasis of the hypothalamic–pituitary-gonadal (HPG) axis. For patients with POI who have fertility demands or concurrent cardiometabolic abnormalities, the potential impact of microbiota-induced metabolic fluctuations on long-term maternal prognosis and offspring development requires careful delineation. Future research should develop precise pharmacokinetic-pharmacodynamic (PK-PD) models in large animal systems and establish longitudinal cohorts with multidimensional endocrine monitoring to define the clinical therapeutic window of these interventions.

### 6.2 Limitations and unresolved questions

Although this review establishes a translational framework for gut microbiota interventions, current evidence remains predominantly preclinical. There is currently insufficient clinical evidence directly establishing a causal relationship between gut dysbiosis and the pathogenesis of human POI. Existing mechanistic data rely heavily on chemotherapy-induced rodent models, ovariectomy-related models, and immortalized cell lines. The translational fidelity of these models to complex human ovarian physiology, such as specific follicular dynamics and reproductive timelines, requires systematic validation. Direct pathological evidence of SIRT1–FoxO1 axis aberrations in granulosa cells from patients with POI is lacking and requires validation in patient-derived ovarian tissue samples or primary cell models. Furthermore, determining the causal nature of microbial alterations at the population level is challenging. Although Mendelian randomization studies have provided preliminary evidence suggesting possible causal links between the microbiota and POI risk (Wang et al., 2023), remodeling of the gut microbiome in patients may merely represent a downstream phenotype triggered by estrogen deprivation, metabolic shifts, or secondary medication rather than an initiating factor in ovarian senescence.

Moreover, the pleiotropic nature of mechanistic networks and the heterogeneity of clinical phenotypes constitute a dual barrier to validating this hypothesis. As a pleiotropic molecule, butyrate exerts its effects through multiple parallel pathways, including HDAC inhibition, activation of receptors such as FFAR and GPR109A, and SIRT1 signaling. This concurrent target responsiveness creates a methodological limitation for precisely defining the relative contribution of the SIRT1–FoxO1 axis *in vivo*. Determining this causal relationship requires cell-type-specific knockout models. It is important to recognize the inherent heterogeneity of POI, which encompasses a broad spectrum of etiological factors, including chromosomal abnormalities, autoimmune inflammation, iatrogenic damage, and idiopathy. The pathogenic weight of gut–ovary axis dysfunction inevitably varies across etiological subtypes, implying that a universal intervention strategy is not applicable. Future research should focus on developing individualized intervention strategies tailored to individual patients, considering their etiology and baseline microbiome stratification.

### 6.3 Prospective research directions

To advance this hypothesis toward clinical translation, future research should address both mechanistic elucidation and clinical evidence. First, establishing temporal causality between butyrate depletion and the pathological progression of POI requires integrating multi-omics data and ovarian reserve markers within prospective longitudinal cohorts. In parallel, single-cell sequencing and spatial transcriptomics should be employed to comprehensively map cell type-specific response profiles of the SIRT1–FoxO1 axis within a highly heterogeneous ovarian microenvironment. In the domain of pharmacological translation, rigorous characterization of the PK-PD properties of butyrate precursors and NAD + supplements in large animal models will be essential for defining safe therapeutic windows. In addition, systematic toxicological and immunogenicity evaluations of novel targeted delivery platforms, such as MSC-EVs, are necessary. The validation of clinical intervention strategies depends on two key factors. First, high-quality RCTs stratified according to baseline gut microbiota are required. Second, drug repurposing trials for SIRT1 modulators with established clinical safety profiles, such as metformin and nicotinamide riboside, should be initiated. Integration of these trials with prospective multidimensional biomarker monitoring may provide decisive clinical evidence for metabolic remodeling therapies for POI.

This review proposes the gut–butyrate–SIRT1–FoxO1 axis as a central mechanistic framework linking gut microbial ecology to ovarian pathophysiology in POI. A systematic review of evidence revealed that depletion of butyrate-producing bacteria in patients with POI may lead to systemic butyrate deficiency, which may disrupt multiple protective pathways in granulosa cells. By linking gut metabolism with ovarian function, this study provides a foundation for translating mechanistic insights into more precise future therapies for POI. However, the current evidence base remains primarily preclinical, and rigorous clinical validation will be required before these approaches can be translated into therapeutic applications.

## Glossary

AMH: Anti-Müllerian hormone
AMPK: AMP-activated protein kinase
ATG3: Autophagy related 3
ATMP: Advanced therapy medicinal product
ATP: Adenosine triphosphate
Bax: BCL2-associated X protein
Bak: BCL2 antagonist/killer 1
CTA: Clinical trial application
FFAR2: Free fatty acid receptor 2
FFAR3: Free fatty acid receptor 3
FMT: Fecal microbiota transplantation
FSH: Follicle-stimulating hormone
FSHR: Follicle-stimulating hormone receptor
FVT: Fecal virome transplantation
FoxO1: Forkhead box protein O1
FOXL2: Forkhead box protein L2
GMP: Good manufacturing practice
GPCR: G protein-coupled receptor
GPX1: Glutathione peroxidase 1
GPR109A: G protein-coupled receptor 109A
HDAC: Histone deacetylase
HIF-1*α*: Hypoxia-inducible factor 1 alpha
HPG axis: Hypothalamic–pituitary–gonadal axis
HRT: Hormone replacement therapy
IL-6: Interleukin 6
IL-8: Interleukin 8
IND: Investigational new drug
LPS: Lipopolysaccharide
MAMs: Mitochondria-associated endoplasmic reticulum membranes
MCT1: Monocarboxylate transporter 1
MOMP: Mitochondrial outer membrane permeabilization
mPTP: Mitochondrial permeability transition pore
MSC-EVs: Mesenchymal stem cell-derived extracellular vesicles
NAD+: Nicotinamide adenine dinucleotide
NF-κB: Nuclear factor kappa B
NLRP3: NLR family pyrin domain containing 3
NMN: Nicotinamide mononucleotide
NR: Nicotinamide riboside
Nrf2: Nuclear factor erythroid 2-related factor 2
PARP: Poly(ADP-ribose) polymerase
PGC-1α: Peroxisome proliferator-activated receptor gamma coactivator 1 alpha
PK–PD: Pharmacokinetic–pharmacodynamic
POI: Premature ovarian insufficiency
RCT: Randomized controlled trial
ROS: Reactive oxygen species
SIRT1: Sirtuin 1
SIRT3: Sirtuin 3
SMCT1: Sodium-coupled monocarboxylate transporter 1
SLC5A8: Solute carrier family 5 member 8
SLC16A1: Solute carrier family 16 member 1
SOD2: Superoxide dismutase 2
TFAM: Mitochondrial transcription factor A
TGR5: Takeda G protein-coupled receptor 5
TLR4: Toll-like receptor 4
TNF-α: Tumor necrosis factor alpha
Tregs: Regulatory T cells
ZO-1: Zonula occludens 1
ΔΨm: Mitochondrial membrane potential
