# Supplementary material for: Gut microbiota-derived butyrate and the SIRT1/FoxO1 axis: epigenetic-metabolic regulation of ovarian function in premature ovarian insufficiency—a comprehensive review
Source: Front Microbiol. 2026 Jul 27;17:1856217. doi: 10.3389/fmicb.2026.1856217 (PMC13454030; doi:10.3389/fmicb.2026.1856217)
Supplement: Supplementary file 1 [file Supplementary_file_1.docx]

Figure S1. PRISMA flow diagram of literature search and study selection. A total of 6,744 records were identified from PubMed (n=6,356) and Web of Science (n=388). After removing 2,244 duplicates, 4,500 records remained for screening. Following title (n=3,300 excluded), abstract (n=800 excluded), and full-text (n=321 excluded) assessment, 79 studies were included in this narrative review.
